# Supplementary material for: Injectable Platelet-Rich Fibrin as a Drug Carrier Increases the Antibacterial Susceptibility of Antibiotic—Clindamycin Phosphate
Source: Int J Mol Sci. 2022 Jul 3;23(13):7407. doi: 10.3390/ijms23137407 (PMC9266531; doi:10.3390/ijms23137407)
Supplement: Supplementary file 1 [file ijms-23-07407-s001.zip › ijms-1783971-supplementary.pdf]

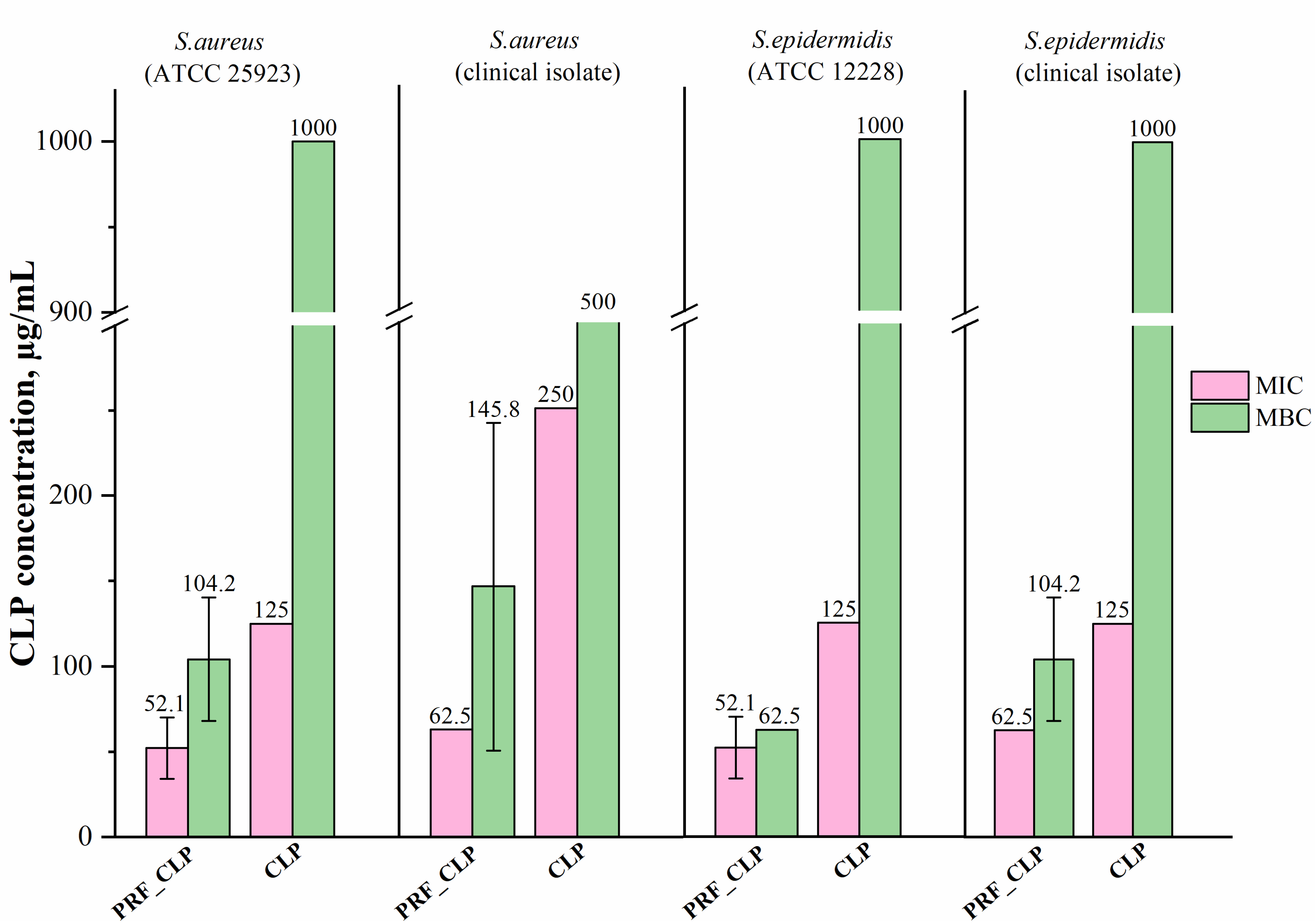

Figure S1. MIC and MBC value differences between CLP and PRF_CLP samples against four bacteria stains (*S.aureus* (ATCC 25923), *S.epidermidis* (ATCC 12228), *S.aureus* (clinical isolate), *S.epidermidis* (clinical isolate). The graphs show the mean MIC and MBC values calculated from the results of antibacterial tests in 3 donors, compared with the MIC and MBC values obtained with pure CLP.

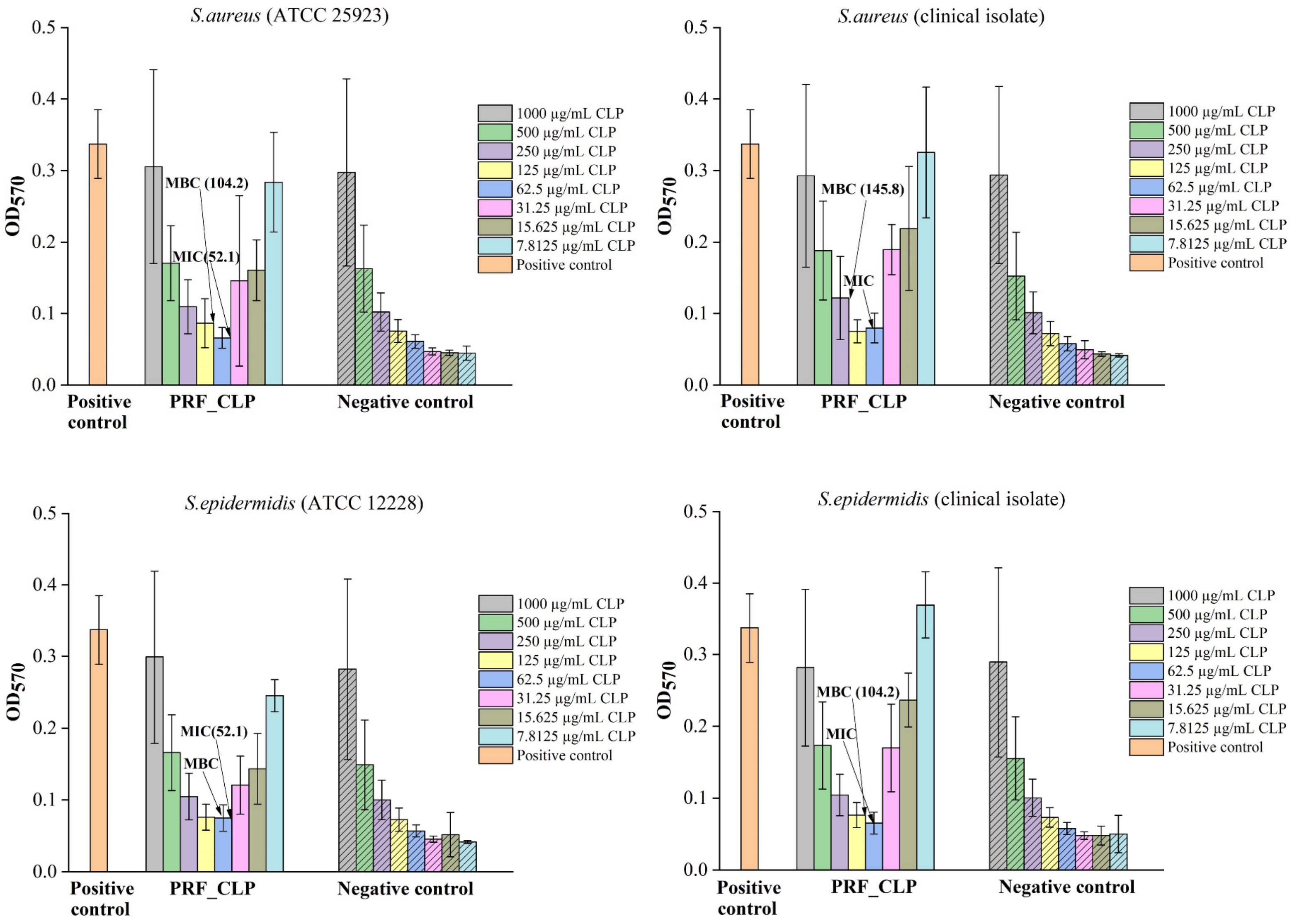

Figure S2. Antibacterial properties of PRF_CLP samples for 4 bacteria strains (*S.aureus* (ATCC 25923), *S.epidermidis* (ATCC 12228), *S.aureus* (clinical isolate), *S.epidermidis* (clinical isolate). The graphs show the mean MIC and MBC values calculated from the results of 3 donor antibacterial tests. Pure bacterial suspension (10^6 CFU/mL) as positive control and pure sterile Mueller-Hinton broth as negative control were used.

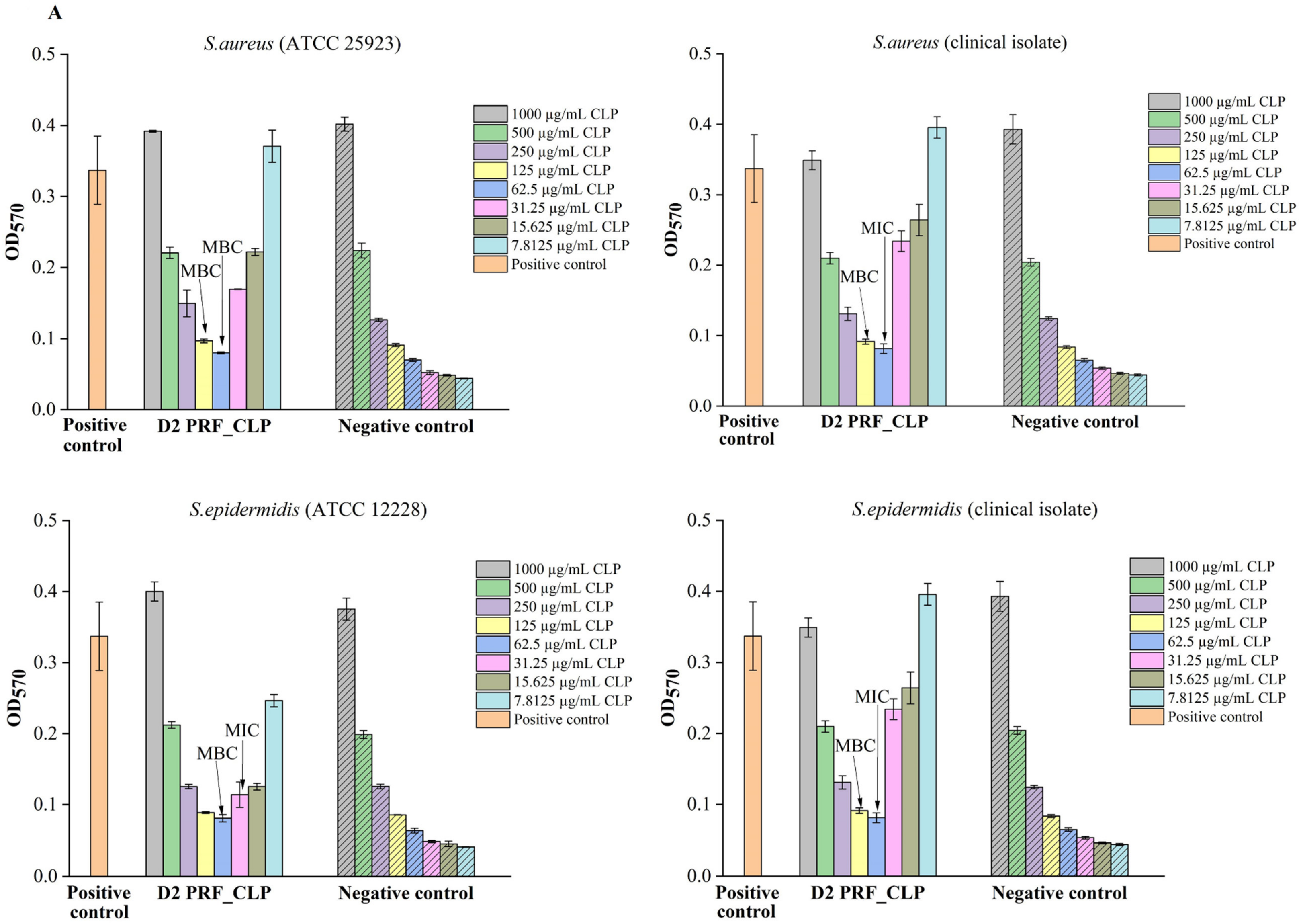
A
S.aureus (ATCC 25923)
S.aureus (clinical isolate)
S.epidermidis (ATCC 12228)
S.epidermidis (clinical isolate)
OD570
0.0
0.1
0.2
0.3
0.4
0.5
Positive control
D2 PRF_CLP
Negative control
MBC
MIC
1000 μg/mL CLP
500 μg/mL CLP
250 μg/mL CLP
125 μg/mL CLP
62.5 μg/mL CLP
31.25 μg/mL CLP
15.625 μg/mL CLP
7.8125 μg/mL CLP
Positive control

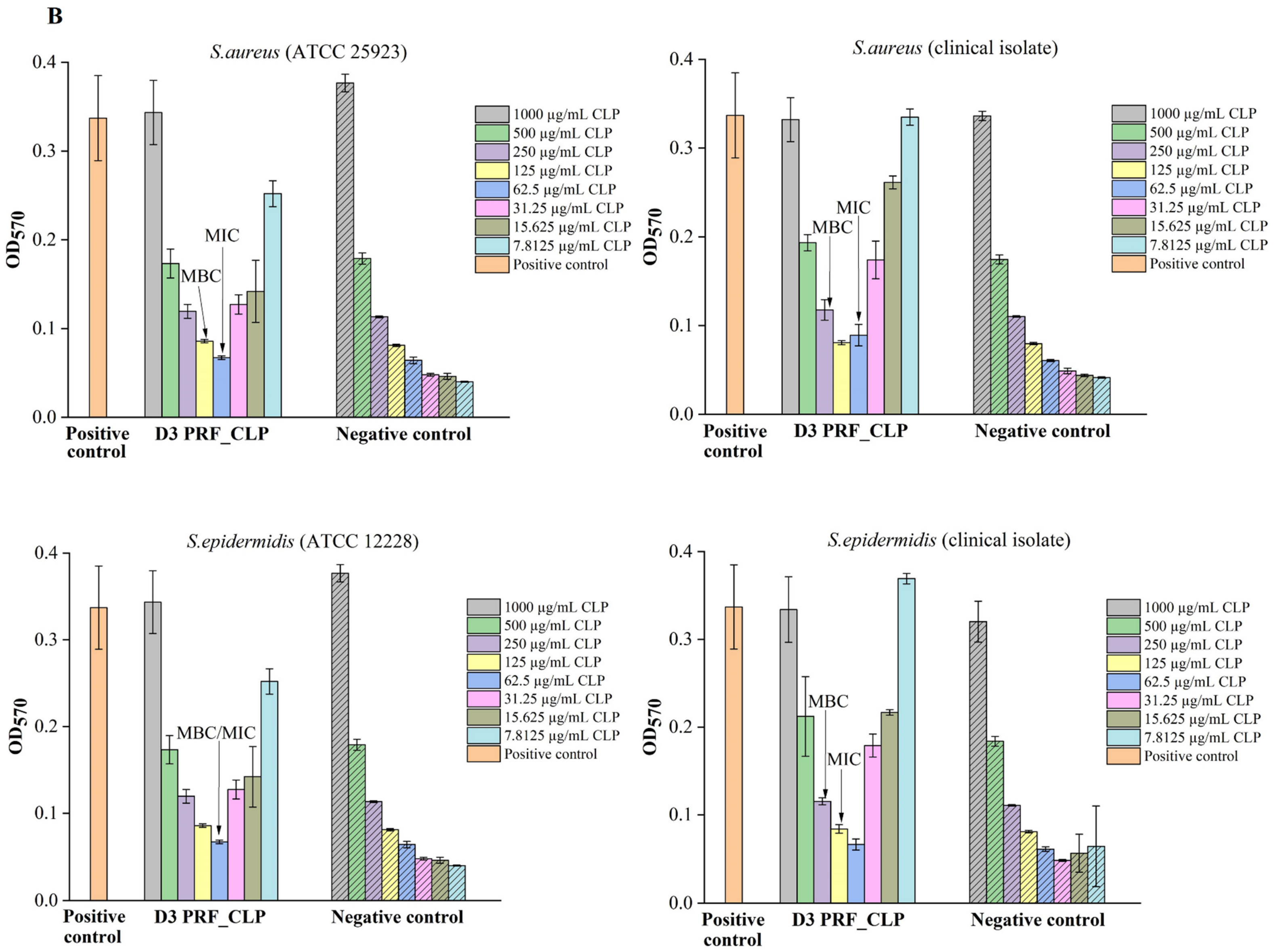

Figure S3. Antibacterial properties of PRF_CLP samples at various concentrations of CLP solution for 4 bacteria strains (*S.aureus* (ATCC 25923), *S.epidermidis* (ATCC 12228), *S.aureus* (clinical isolate), *S.epidermidis* (clinical isolate) : A) For PRF_CLP samples prepared from donor 2 blood; B) For PRF_CLP samples prepared from donor 3 blood. Pure bacterial suspension (10^6 CFU/mL) as positive control and pure sterile Mueller-Hinton broth as negative control were used.
